# Supplementary material for: An empirical analysis based on a panel threshold model of the effect of Internet development on the efficiency of Chinese government public service supply
Source: PLoS One. 2022 Jul 13;17(7):e0271390. doi: 10.1371/journal.pone.0271390 (PMC9278768; doi:10.1371/journal.pone.0271390)
Supplement: Attachment — Submitted filename: Response to Reviewers.docx [file pone.0271390.s002.docx]

Response to Reviewers

We sincerely appreciate your valuable and constructive comments on our manuscript (Manuscript Number:PONE-D-22-13139). We have studied the comments carefully and have made revisions. According to these comments, we have revised our manuscript very carefully. All changed parts are highlighted in yellow in the revised manuscript. The point-by-point answers to all comments are listed as follows.

**The following are Journal Requirements.**

**Point 1**: Please ensure that your manuscript meets PLOS ONE's style requirements, including those for file naming.

Response 1: Thank you for your comments. In the revised version, we have checked and modified the full text of the manuscript according to the PLOS ONE's style requirements.

**Point 2**: Please remove any funding-related text from the manuscript and let us know how you would like to update your Funding Statement.

Response 2: Thank you for your comments. In the revised version, we have deleted the text related to funds*(line 481-482)*. At the same time, Our Funding Statement reads remains as follows: This research was supported by the National Social Science Fund of China [grant number 20VYJ027].The funders had no role in study design, data collection and analysis, decision to publish, or preparation of the manuscript.

**Point 3**: In your Data Availability statement, you have not specified where the minimal data set underlying the results described in your manuscript can be found. PLOS defines a study's minimal data set as the underlying data used to reach the conclusions drawn in the manuscript and any additional data required to replicate the reported study findings in their entirety.

Upon re-submitting your revised manuscript, please upload your study’s minimal underlying data set as either Supporting Information files or to a stable, public repository and include the relevant URLs, DOIs, or accession numbers within your revised cover letter.

Response 3: Thank you for your comments. Upon re-submitting our revised manuscript, We have uploaded our study’s minimal underlying data set as either Supporting Information files and explained it in the revised manuscript *(line 608-609)*.

**Point 4:**Please ensure that you have an ORCID iD and that it is validated in Editorial Manager.

Response 4: Thank you for your comments. I have completed the Authorization process and have been logged in to Editorial Manager. My ORCID iD was retrieved securely from ORCID and successfully linked to my user record.

**Point 5:**Please review your reference list to ensure that it is complete and correct.

Response 5: Thank you for your comments. In the revised version, we have checked the list of all references in the manuscript to ensure that it is complete and correct

**The following are Additional Editor Comments.**

**Point 1**:Dear authors, I am sharing the observations of the reviewers, which are minimal; however, I would like the number of references to increase. I suggest the following.

[1] Blanco-Orozco, N., Arce-Díaz, E., & Zúñiga-Gonzáles, C. (2015). Integral assessment (financial, economic, social, environmental and productivity) of using bagasse and fossil fuels in power generation in Nicaragua. Revista Tecnología en Marcha, 28(4), 94-107. DOI 10.18845/tm.v28i4.2447 https://publons.com/publon/32281799/

[2] Zuniga González, C. A. (2020). Total factor productivity growth in agriculture: Malmquist index analysis of 14 countries, 1979-2008. REICE: Revista Electrónica De Investigación En Ciencias Económicas, 8(16), 68–97. https://doi.org/10.5377/reice.v8i16.10661

[3] Zuniga-Gonzalez, Carlos Alberto (2021), “Total factor productivity in the INTA Chinandega rice variety”, Mendeley Data, V2, doi: 10.17632/76m7p7mvsg.2 https://data.mendeley.com/datasets/76m7p7mvsg/2

[4] González, C. A. Z. (2011). Technical efficiency of organic fertilizer in small farms of Nicaragua: 1998-2005. African Journal of Business Management, 5(3), 967-973. https://publons.com/publon/11272633/

[5] Dios-Palomares, R., Alcaide, D., Diz, J., Jurado, M., Prieto, A., Morantes, M., & Zúñiga, C. A. (2015). Analysis of the efficiency of farming systems in Latin America and the Caribbean considering environmental issues. Revista Cientifica, Facultad de Ciencias Veterinarias, Universidad del Zulia, 25(1), 43-50. https://publons.com/publon/3106827/

[6] Bermúdez-León, D. S., & Zúniga-González, C. A. (2016). Information and communication technologies (ICT) as a response to educational needs in rural areas in Nicaragua. Rev. Iberoam. Bioecon. Cambio Clim., 2(4), 563–574. <https://doi.org/10.5377/ribcc.v2i4.5931>

Response 1: Thank you for your comments. In the revised version, we have added the above references*([1]:line 119,line557-560; [2]:line 223,line584-587;* *[3]:line 223,line587-589 ; [4]:line 223,line590-591 ; [5]:line 223,line592-595 ; [6]:line 60,line507-510;).*

**The following are the comments and suggestions pointed out by Reviewers.**

**Point 1**: Authors change the title of the manuscript “An empirical analysis based on a panel threshold model of Chinese government public service supply with internet development efficiency”.

Response 1: Thank you for your comments. In the revised version, we have revised the title of the article as “An empirical analysis based on a panel threshold model of the Effect of Internet Development on the Efficiency of Chinese Government Public Service Supply”*(line 1-4)*.

**Point 2:** Authors draw a graphical abstract of the model.

Response 2: Thank you for your comments. In the revised version, we have supplemented the likelihood ratio function graphs of the two thresholds (Fig. 1 and Fig. 2), and further explained the contents of the two graphs *(line 318-326)*. The pictures will be uploaded separately in TIFF format.

**Point 3:** Proofread the entire manuscript.

Response 3: Thank you for your comments. In the revised version, we have carefully Proofread the full text of the manuscript.
